# The α-mating factor secretion signals and endogenous signal peptides for recombinant protein secretion in *Komagataella phaffii*

**DOI:** 10.1186/s13068-022-02243-6

**Published:** 2022-12-16

**Authors:** Chenwei Zou, Lingfang Lu, Shengyan Wang, Chenshan Zhang, Xuequn Chen, Yao Lin, Yide Huang

**Affiliations:** 1 Provincial University Key Laboratory of Cellular Stress Response and Metabolic Regulation, College of Life Sciences, Fujian Normal University, Fuzhou, 350007 China; 2 Engineering Research Center of Industrial Microbiology, College of Life Sciences, Fujian Normal University, Fuzhou, 350007 China; 3 Central Laboratory at the Second Affiliated Hospital of Fujian Traditional Chinese Medical University, Innovation and Transformation Center, Fujian University of Traditional Chinese Medicine, Fuzhou, 350122 Fujian People’s Republic of China

**Keywords:** *Komagataella phaffii*, α-Mating factor, Peptide signal, Protein secretion

## Abstract

**Background:**

The budding yeast *Komagataella phaffii* (*Pichia pastoris*) is widely employed to secrete proteins of academic and industrial interest. For secretory proteins, signal peptides are the sorting signal to direct proteins from cytosol to extracellular matrix, and their secretion efficiency directly impacts the yields of the targeted proteins in fermentation broth. Although the α-mating factor (MF) secretion signal from *S. cerevisiae*, the most common and widely used signal sequence for protein secretion, works in most cases, limitation exists as some proteins cannot be secreted efficiently. As the optimal choice of secretion signals is often protein specific, more secretion signals need to be developed to augment protein expression levels in *K. phaffii*.

**Results:**

In this study, the secretion efficiency of 40 α-MF secretion signals from various yeast species and 32 endogenous signal peptides from *K. phaffii* were investigated using enhanced green fluorescent protein (EGFP) as the model protein. All of the evaluated α-MF secretion signals successfully directed EGFP secretion except for the secretion signals of the yeast *D. hansenii* CBS767 and *H. opuntiae*. The secretion efficiency of α-MF secretion signal from *Wickerhamomyces ciferrii* was higher than that from *S. cerevisiae*. 24 out of 32 endogenous signal peptides successfully mediated EGFP secretion. The signal peptides of chr3_1145 and FragB_0048 had similar efficiency to *S. cerevisiae* α-MF secretion signal for EGFP secretion and expression.

**Conclusions:**

The screened α-MF secretion signals and endogenous signal peptides in this study confer an abundance of signal peptide selection for efficient secretion and expression of heterologous proteins in *K. phaffii*.

**Supplementary Information:**

The online version contains supplementary material available at 10.1186/s13068-022-02243-6.

## Introduction

*Komagataella phaffii* (also referred to as *Pichia pastoris*) is a methylotrophic yeast, which can utilize methanol as sole carbon and energy source. After its failure as single-cell protein (SCP) production, *K. phaffii* was subsequently developed into a heterologous protein expression host for the production of recombinant proteins [1]. Over the past 30 years, *K. phaffii* has become one of the most popular expression hosts attributed to its various advantages: its ability to reach high cell densities on defined media, the presence of strong and tightly methanol-regulated Alcohol Oxidase I (*AOX1*) promoter, high protein expression levels and low incidence of hyperglycosylation. More than 5000 heterologous proteins have been reported to be successfully expressed in the *K. phaffii* system [2]. The recombinant proteins expressed in *K. phaffii* involved in industrial enzymes, vaccine, antibody fragments, cytokines and membrane proteins [3–8].

The heterologous proteins expressed in *K. phaffii* are generally secreted into the culture medium. One of the important reasons is that *K. phaffii* has a secretory pathway consisted of the endoplasmic reticulum (ER) and Golgi apparatus to ensure proper protein folding, processing and modification including disulfide bond formation, glycosylation and oligomerization. Compared to the secretory pathway of *S. cerevisiae*, the secretory pathway of *K. phaffii* are more similar to that of higher eukaryotes in having stacked Golgi cisternae [9–11]. Secretory proteins are released to the extracellular medium as the soluble forms, which are more similar to the native proteins in structure and have higher physiological activity. Another reason is that *K. phaffii* secretes few endogenous proteins out of the cell, which facilitates the purification of recombinant proteins [12].

For secretory proteins, signal peptides are the sorting signal to direct proteins from cytosol to extracellular matrix [13, 14]. To produce the recombinant proteins in expression systems, the secretion efficiency of signal peptides directly impacts the yields of the targeted proteins in fermentation broth [15]. The α-mating factor (MF) secretion signal from *S. cerevisiae* is the most common and widely used signal sequence for recombinant protein secretion in *K. phaffii*. The α-MF secretion signal of *S. cerevisiae* consists of 85 amino acids and contains two regions: a pre-peptide (signal peptide) consisting of N-terminal 19 amino acids and a pro-peptide consisting of 66 amino acids from position 20 to 85 [16]. Pre-peptide mediates targeting the secretory proteins into the endoplasmic reticulum, and pro-peptide is believed to be involved in mediating secretory proteins into endoplasmic reticulum-derived COPII transport vesicles and enhances secretion efficiency of recombinant proteins [17, 18]. Although α-MF secretion signal of *S. cerevisiae* has been successfully used for the secretion of a large number of heterologous proteins in *K. phaffii*, some proteins were unsuccessfully expressed when using the α-MF secretion signal [19]. In recent years, endogenous signal peptides of *K. phaffii* were developed to mediate secretion of heterologous proteins. Several endogenous signal peptides were reported to yield much more efficient secretion than α-MF secretion signal of *S. cerevisiae* [20, 21]*.*

In addition to *S. cerevisiae*’s, 39 α-MF genes from other yeast species can be found in the NCBI database. It is unknown whether their α-MF secretion signal can also efficiently mediate protein secretion in *K. phaffii* so far. After sequencing of the *K. phaffii* genome in 2009, Schutter et al. analyzed signal sequences of *K. phaffii* according to the homologs of functionally annotated secreted proteins in *S. cerevisiae* and revealed a multitude of endogenous signal peptides [22], which can allow screening high efficiency secretion signals for augmenting protein expression levels in *K. phaffii*. In this study, we systematically evaluated secretion efficiency of 40 α-MF secretion signals from various yeast species and 32 endogenous signal peptides from *K. phaffii* with a D-score≥ 0.95 using EGFP as the model protein.

## Results

### Protein secretion with the α-MF secretion signals from *S. cerevisiae*, *K. phaffii* and *K. lactis*

**Fig. 1:**
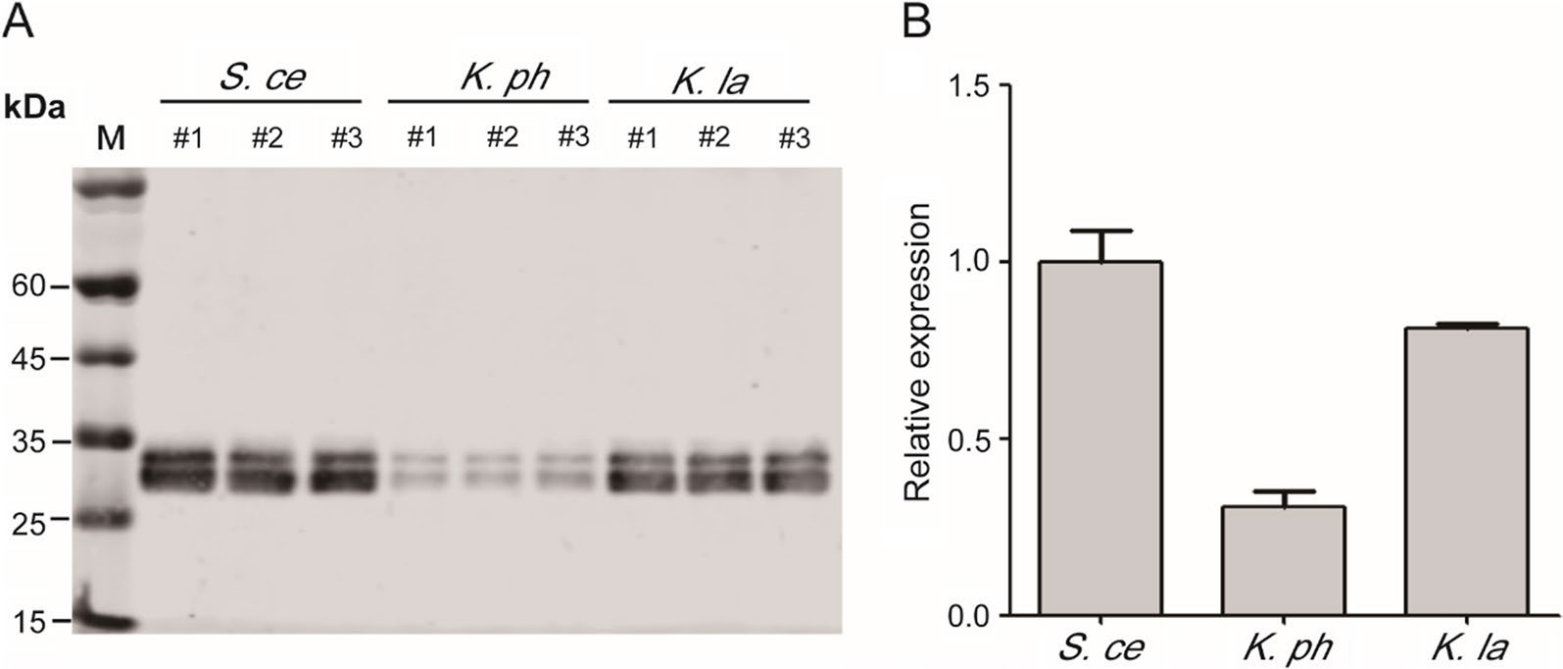
Secretion and expression of EGFP mediated by the α-MF secretion signals from *S. cerevisiae*, *K. phaffii* and *K. lactis.* A: The expression analysis of recombinant EGFP by Western blot using anti-EGFP primary antibody. B: The quantitative analysis to A. #1, #2 and #3 represent three different recombinant strains. M: protein marker; *S. ce*: *S. cerevisiae; K. ph*: *K. phaffii*; *K. la*: *K. lactis*

The secretion of most proteins produced in *K. phaffii* is mediated by the α-MF secretion signal from *S. cerevisiae*. In the yeast *Kluyveromyces lactis* expression system (New England BioLabs Inc.), *K. lactis* α-MF secretion signal, not *S. cerevisiae* α-MF secretion signal, is employed to secrete recombinant proteins. It is possible that the α-MF secretion signal from *K. lactis* works better than that from *S. cerevisiae* in *K. lactis* cells. The genome sequence from *K. phaffii* reveals a α-MF gene in *K. phaffii* GS115 strain. The α-MF secretion signal from *S. cerevisiae* works in most cases in *K. phaffii,* although there have been no studies to compare it to α-MF secretion signals from other yeast species. Using EGFP as a reporter, the secretion efficiency of the three α-MF secretion signals in *K. phaffii* were compared. The results showed that the secretion efficiency of *S. cerevisiae* α-MF secretion signal is the highest followed by *K. lactis*’s and *K. phaffii*’s (Fig. 1), indicating that the secretion efficiencies of α-MF secretion signals from different yeast species on protein expression were different in *K. phaffii* system.

### Evaluation of α-MF secretion signals on the effect of protein secretion

**Fig. 2:**
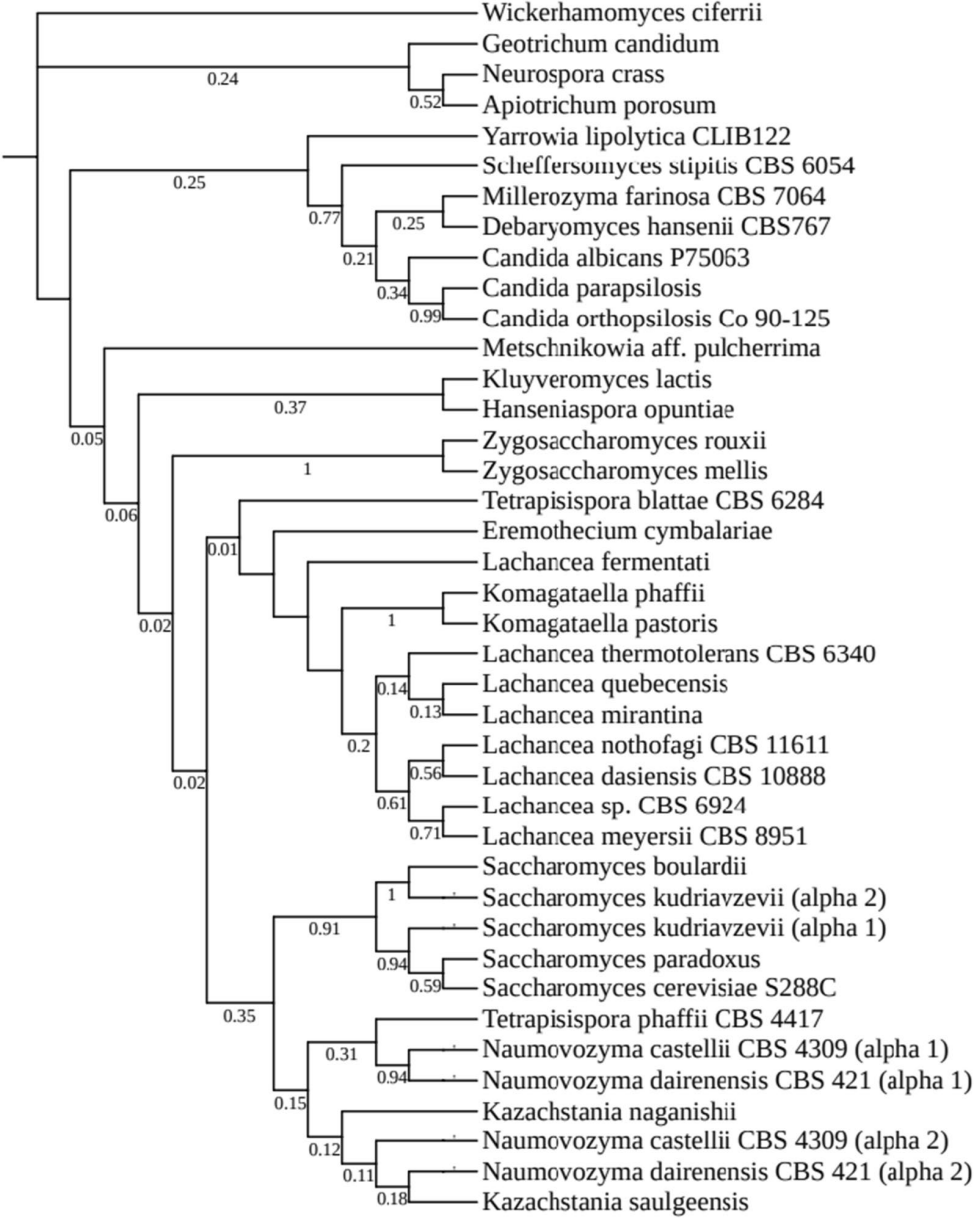
Phylogenetic tree of the α-MF precursors. The tree was constructed using the amino acid sequences of α-MF precursors. The numbers at the forks indicate the bootstrap confidence values

Searching NCBI database, 40 α-MF genes from different yeast species including *S. cerevisiae*, *K. lactis* and *K. phaffii* were found (Additional file 1: Table S1). Whether their secretion signals also work well like *S. cerevisiae*’s in *K. phaffii* has not been evaluated. The α-MF precursors were used to construct a phylogenetic tree (Fig. 2). The constructed phylogenetic tree showed several distinct clusters of α-MF precursors in yeasts. A highly close relation between *K. pastoris* and *K. phaffii* was revealed from the phylogenetic tree.

**Fig. 3:**
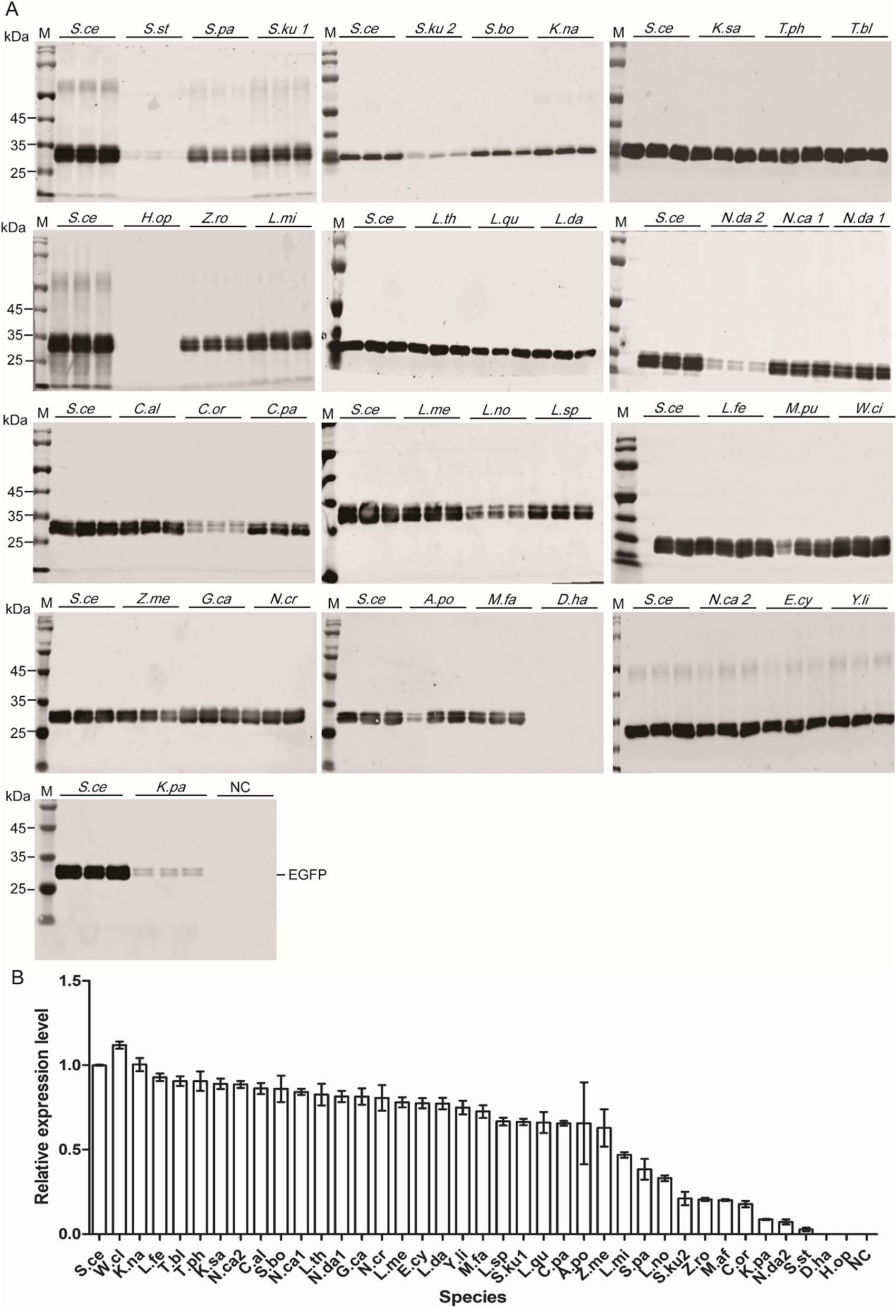
The secretion and expression of EGFP mediated by α-MF secretion signals from different yeast species. **A** The expression analysis of recombinant EGFP by Western blot. **B** The quantitative analysis to A. M: protein marker; *S.ce: S. cerevisiae*; *W.cl*: *W. ciferrii*; *K.na*: *K. naganishii*; *L.fe*: L. fermentati; *T.bl*: *T. blattae* CBS 6284; *T.ph*: *T. phaffii* CBS 4417; *K.sa*: *K. saulgeensis*; *N.ca*2: *N. castellii* CBS 4309 (alpha 2); *C.al*: *C. albicans* P75063; *S.bo*: *S. boulardii*; *N.ca1*: *N. castellii* CBS 4309 (alpha 1); *L.th: L. thermotolerans* CBS 6340; *N.da1*: *N. dairenensis* CBS 421 (alpha 1); *G.ca*: *G. candidum*; *N.cr*: *N. crass*; *L.me*: *L. meyersii* CBS 8951; *E.cy*: *E. cymbalariae*; *L.da*: *L. dasiensis* CBS 10888; *Y.li*: *Y. lipolytica* CLIB122; *M.fa*: *M. farinosa* CBS 7064; *L.sp*: *Lachancea sp.* CBS 6924; *S.ku*1: *S. kudriavzevii* (alpha 1); *L.qu*: *L. quebecensis*; *C.pa*: *C. parapsilosis*; *A.po*: *A. porosum*; *Z.me*: *Z. mellis*; *L.mi*: *L. mirantina*; *S.pa*: *S. paradoxus*; *L.no: L. nothofagi* CBS 11611; *S.ku2: S. kudriavzevii* (alpha 1); *Z.ro*: *Z. rouxii*; *M.pu*: *M. aff. pulcherrima*; *C.or*: *C. orthopsilosis* Co 90-125; *K.pa*: *K. pastoris*; *N.da*2: *N. dairenensis* CBS 421 (alpha 2); *S.st*: *S. stipitis* CBS 6054; *D.ha: D. hansenii* CBS767; H.op: *H. opuntiae*; NC: negative control. Three independent strains were used to evaluate EGFP expression

In order to eliminate codon bias on the effect of translation efficiency when these α-MF secretion signals were used to mediate EGFP secretion, coding sequences of α-MF secretion signals were optimized according to the method established in previous study (Additional file 1: Table S2) [23]. The secretion efficiencies of α-MF secretion signals were evaluated using *S. cerevisiae* α-MF secretion signal as a control. Almost all of the evaluated α-MF secretion signals successfully mediated EGFP secretion. Only the α-MF secretion signals of *D. hansenii CBS767* and *H. opuntiae* failed to mediate EGFP secretion. Except for *W. ciferrii* α-MF secretion signal, the secretion efficiency of other α-MF secretion signals to EGFP was lower than that of *S. cerevisiae* (Fig. 3). The 3-D structures of α-MF secretion signals were predicted using Alphafold 2.0 AI system [24]. Most of α-MF secretion signals showed a conservative structure with a 2-stranded anti-parallel β-sheet followed by an α-helix on C-terminus (Additional file 2: Fig. S2).

### Secretion and expression of EGFP mediated by endogenous signal peptides

**Fig. 4:**
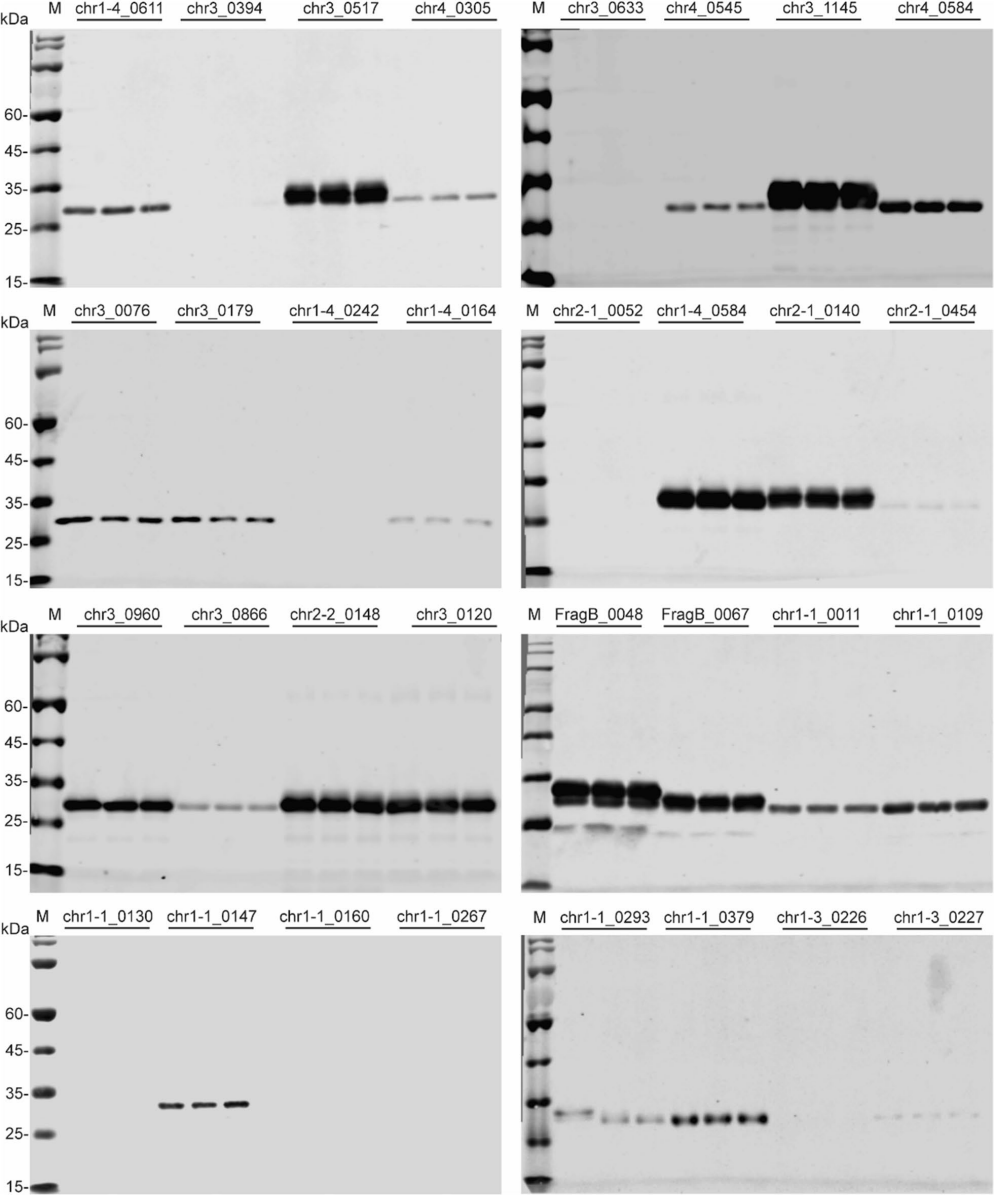
The secretion and expression of EGFP mediated by endogenous signal peptides. The expression of recombinant EGFP was detected by Western blot. M: protein marker. Three independent strains were used to evaluate EGFP expression

After sequencing *K. phaffii* genome in 2009, the genome sequence revealed a total of 54 endogenous signal peptides, which derived from homologs of functionally annotated secretory proteins of *S. cerevisiae* [22]. These predicted endogenous signal peptides will allow screening for functional signal peptides in *K. phaffii*. The D-score of 54 endogenous signal peptides were analyzed using SignalP 5.0. In this study, 32 endogenous signal peptides with D-score values greater than or equal to 0.95 were selected to evaluate EGFP secretion (Additional file 1: Table S3). 24 out of 32 endogenous signal peptides successfully mediated EGFP secretion, and the signal peptides of chr3_0517, chr3_1145, chr1-4_0584, chr2-1_0140, chr3_0960, chr2-2_0148, chr3_0120, FragB_0048 and FragB_0067 directed strong EGFP secretion and expression (Fig. 4).

### Comparison of endogenous signal peptides with *S. cerevisiae* α-MF secretion signal on EGFP secretion

**Fig. 5:**
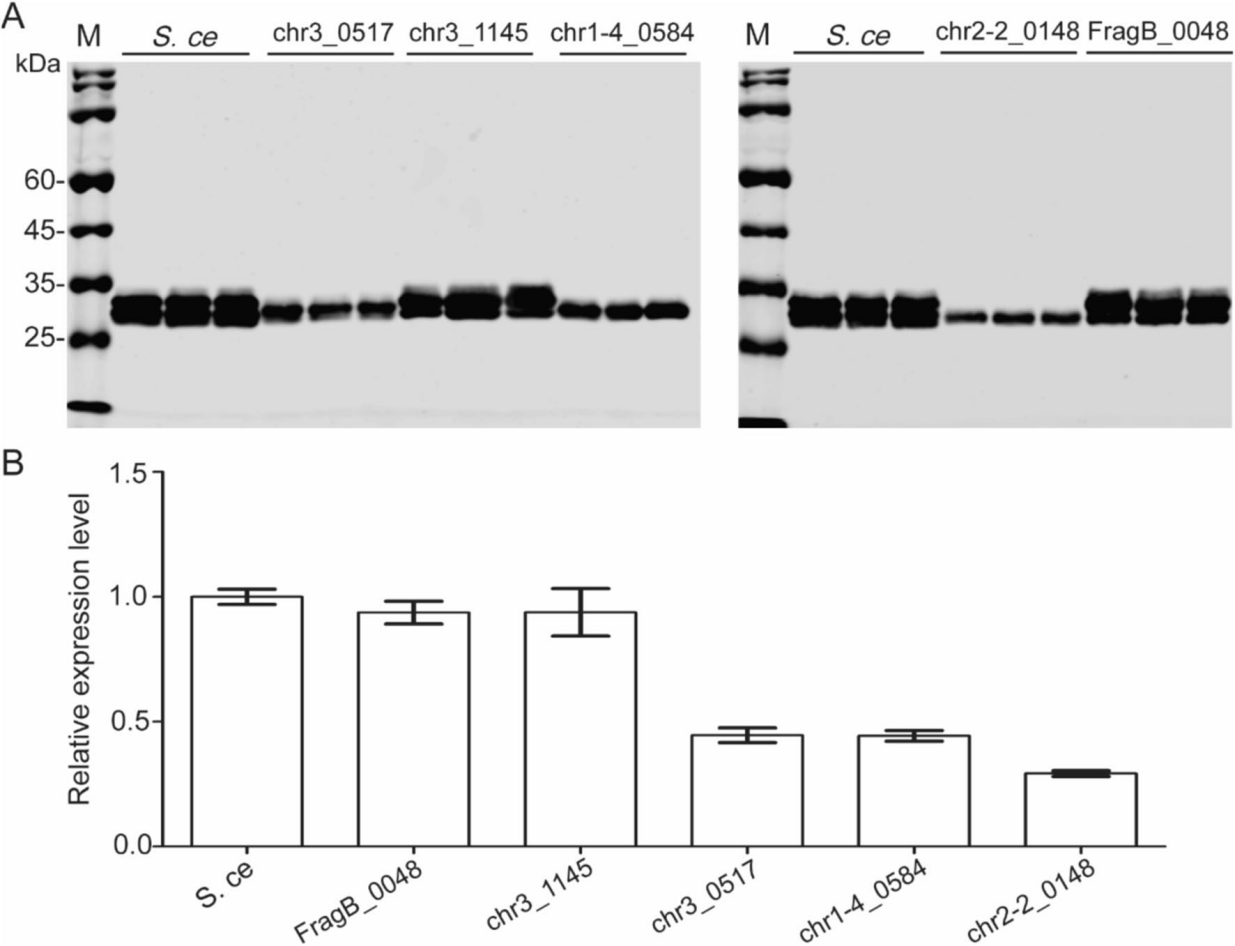
Comparison of endogenous signal peptides with *S. cerevisiae* α-MF secretion signal on EGFP expression. **A** The expression analysis of recombinant EGFP by Western blot. **B** The quantitative analysis to A. M: protein marker; *S. ce*: *S. cerevisiae*. Three independent strains were used to evaluate EGFP expression

The *S. cerevisiae* α-MF secretion signal is the most used signal sequence and has high efficiency for protein secretion. In this study, several endogenous signal peptides with high secretion efficiency were successfully screened (Fig. 4). Whether did these endogenous signal peptides perform better on protein secretion than *S. cerevisiae* α-MF secretion signal? Five endogenous signal peptides with the highest secretion efficiency were selected to compare with *S. cerevisiae* α-MF secretion signal for expressing EGFP. The results showed that signal peptides of chr3_1145 and FragB_0048 had similar efficiency to *S. cerevisiae* α-MF secretion signal for EGFP secretion and expression (Fig. 5).

## Discussion

In this study, 40 α-MF secretion signals from different yeast species were tested for secretion expression of EGFP in *K. phaffii*. 38 out of 40 α-MF secretion signals successfully directed the secretion of EGFP, suggesting that their secretory pathways appear to be conservative in the yeast family. Yeasts are outstanding hosts to produce recombinant proteins for industrial or medical applications [25]. The yeasts including *S. cerevisiae*, *K. phaffii*, *H. polymorpha*, *Y. lipolytica*, *A. adeninivorans*, *K. lactis*, and *S. pombe* are commonly employed as expression hosts for production of recombinant proteins [25]. Few secretion signals have been developed for use in these yeasts. The frequently used signal sequence is the *S. cerevisiae* α-MF secretion signal. The α-MF secretion signals developed in this study will greatly enrich the selection of signal sequences for these yeast expression systems. At the same time, the secretion efficiency of α-MF secretion signal from *W. ciferrii* was higher than that from *S. cerevisiae*, suggesting it can be used to substitute α-MF secretion signal from *S. cerevisiae* for promoting secretion of heterologous proteins in *K. phaffii*.

The α-MF secretion signal contains two regions: a pre-peptide followed by a pro-peptide. The pre-peptide helps the nascent protein translocate to the ER. The pro-peptide is believed to play a significant role in secretion efficiency [26, 27]. The mutation or deletion on pro-peptide of *S. cerevisiae* α-MF changed secretion efficiency of reporter proteins [26, 28]. The deletion of *K. pastoris* pro-peptide significantly increased secretion of reporter proteins in our study (data not shown). The pro-peptide of *S. cerevisiae* α-MF with 66 amino acids forms a certain secondary structure. Lin-cereghino et al. predicted the secondary structure of *S. cerevisiae* α-MF pro-peptide based on a Jpred secondary structure program and knob–socket modeling of tertiary structure. The pro-peptide is consisted of a large loop region framed by two interacting helices [28]. Based on the analysis of the circular dichroism, Chahal et al. released a new structure model of *S. cerevisia*e α-MF pro-peptide with five beta strands and one alpha helix [26]. In this work, we used AphaFold2 model to predict the 3-D structure of α-MF pro-peptides [24]. The structure of *S. cerevisiae* α-MF pro-peptide is consisted of a 2-stranded anti-parallel β-sheet followed by an α-helix on C-terminus (Additional file 2: Fig. S2). Amino acids 50–56 and 60–67 constitute two β-sheet while amino acids 68–78 are present in an α-helix. Studies showed that deletion of amino acids 57–70 located within the secondary structure of *S. cerevisiae* α-MF pro-peptide increased secretion of recombinant protein [27, 28]. Most of α-MF pro-peptides from various yeasts have the same secondary structure like *S. cerevisiae* α-MF pro-peptide, indicating the structure possibly plays a functional role in expression regulation of α-MF pheromone in yeasts.

Although *S. cerevisiae* α-MF secretion signal works in most cases, the native signal peptides from heterologous proteins or endogenous signal peptides from *K. phaffii* are another viable option [20, 21, 29, 30]. Several studies showed that endogenous signal peptides were found to exhibit high secretory activity to reporter proteins [20, 21, 31]. After sequencing the genome of *K. phaffii*, a multitude of endogenous signal sequences were revealed [22]. Few of them have been experimentally tested to mediate secretion of target proteins. In this study, 32 endogenous signal peptides were evaluated for secretory activity, and 24 out of 32 endogenous signal peptides successfully directed EGFP secretion and expression. As the optimal choice of signal peptides is often protein specific, testing different signal peptides should influence overall yield. These endogenous signal peptides provide an abundance of choices for efficient secretion and expression of heterologous proteins in *K. phaffii* system. The α-MF secretion signal mediates posttranslational translocation across the ER membrane, so recombinant proteins that can fold in the cytosol may be inefficiently translocated and thus poorly secreted [32]. Barrero et al. used the peptide signal of *OST1* gene and α-MF pro-peptide from *S. cerevisiae* to engineer a hybrid secretion signal, which yielded efficient secretion for proteins that can fold in the cytosol and for oligomeric proteins [18]. The α-MF secretion signals and endogenous signal peptides screened out in this study can also be used to construct the hybrid secretion signal library for secretion of heterologous proteins which can fold or oligomerize in the yeast cytosol.

## Conclusions

In this study, the secretion efficiency of 40 α-MF secretion signals from various yeast species and 32 endogenous signal peptides from *K. phaffii* were evaluated. Thirty-eight α-MF secretion signals and 24 endogenous signal peptides successfully mediated the secretion and expression of the reporter protein. The screened α-MF secretion signals and endogenous signal peptides can allow screening for the optimal signal-ORF combination, which may result in augmented protein expression levels in *K. phaffii*.

## Materials and methods

### Plasmids, strains and culture media

The plasmid pPIC9K was used as backbones to construct expression vectors containing different signal sequences for secretory expression. *E. coli* Top10′ was used as a host for genetic manipulation and *K. phaffii* GS115 strain was employed as a host for expression of heterologous proteins. Luria–Bertani (LB) liquid medium (0.5% yeast extract, 1% Peptone, and 0.5% NaCl) with 100 μg/mL ampicillin was used in the growth of bacteria. GS115 strain was grown in YPD agar plates (1% yeast extract, 2% peptone, 2% dextrose and 1.5% agar). MD agar (2% dextrose, 1.34% yeast nitrogen base, 4 × 10^−5^% biotin and 1.5% agar) were used as selected medium to screen transformants integrated into pPIC9K plasmids. BMGY [1% yeast extract, 2% tryptone, 1.34% YNB, 1% glycerol, 100 mM potassium phosphate (pH 6.0) and 4 × 10^−5^% biotin] and BMMY [1% yeast extract, 2% tryptone, 1.34% YNB, 1% methanol, 100 mM potassium phosphate (pH 6.0)] media were employed to express the reporter. Top10′ was cultured at 37 ℃ and GS115 at 30 ℃ with stirring rate 220 rpm in a shaker.

### The selection for α-MF secretion signals and endogenous signal peptides

For collecting the information of α-MF secretion signals, we searched NCBI protein database using “alpha mating factor” as the key word. The results of this search were analyzed using the Protein Blast tool of NCBI to filter out the identical protein sequences from different species. The collected protein sequences were further evaluated using SignalP 5.0 software to confirm that there is a signal peptide in the sequence.

According to the homology of functionally annotated secretory proteins of *S. cerevisiae*, De Schutter et al. analyzed the genome sequence of *K. phaffii* and revealed a total of 54 endogenous signal peptides in *K. phaffii* [22]*.* The D-score of 54 endogenous signal peptides were analyzed using SignalP 5.0. In this study, 32 endogenous signal peptides with D-score values greater than or equal to 0.95 were selected to evaluate the reporter secretion.

### Construction of expression vectors

To evaluate the α-MF secretion signals from different yeasts and endogenous signal peptides from *K. phaffii* on the effect of protein secretion, The *EGFP* was used as the reporter gene. The *EGFP* was amplified from pEGFP-N1 plasmid by PCR and cloned into the pPIC9K expression vector between *Sna*B I and *Eco*R I sites for construction of pPIC9K-EGFP. The coding sequences of α-MF secretion signal and endogenous signal peptides were synthesized by gene company (Wuhan GeneCreate Biological Engineering Co., Ltd.) and cloned into the *Bam*H I and *Sna*B I restriction sites of pPIC9K-EGFP, keeping the secretion signal and endogenous signal peptide coding sequence with EGFP gene in the same reading frame (Additional file 2: Fig. S1).

### Electroporation of *K. phaffii*

Electroporation of plasmids into *K. phaffii* were performed as described previously [33]. Briefly, the purified plasmids were digested with restriction enzyme recommended by Pichia Expression Kit manual to obtain linear DNA. The 5–10 μg of linear plasmid DNA was used for electroporation. The transformed cells were spread on MD agar plates. The plates were incubated at 29 ℃ for 2–3 days until colonies appeared.

### EGFP expression

Three colonies from MD plates were picked and cultured in BMGY at 29 ℃ at 220 rpm broth in a shaking incubator until the culture reaches an OD_600_ = 2–4. Then, the cells were harvested by centrifuging at 3000 ×*g* for 5 min at room temperature. The cell pellet was resuspended to an OD_600_ of 1.0 in BMMY medium with 1% methanol. The cells were cultured at 29 ℃ for 72 h and added 100% methanol to a final concentration of 1% methanol every 24 h to maintain induction. Centrifugation was performed to collect the supernatant at 12,000 ×*g* at 4 ℃ for 10 min. the supernatant was stored at – 80 ℃ until ready to assay.

### Western blot

The expression of EGFP was evaluated by Western blot. The 10 μL of supernatant was loaded into each well of 10% SDS-PAGE gel. After finishing the electrophoresis, the proteins in the gel were transferred to Hybond-C nitrocellulose membrane (Amersham Bioscience). The transfer was done at 100 V for 2 h. Anti-EGFP antibody (Proteintech, China, Cat no. 50430-2-AP) and IRDye 800CW-conjugated goat anti-rabbit secondary antibodies (LI–COR Biosciences, Lincoln, NE, USA; cat. no. C60607-15) were employed as the primary and secondary antibody, respectively. The hybridization signals were detected and measured using LICOR Odyssey system (LI–COR, Nebraska, USA).

### Phylogenetic analysis

In the phylogenetic analysis, the amino acids sequences of α-MF were aligned using MUSCLE, and the Maximum Likelihood (ML) tree was constructed by MEGA X, bootstrap was set to 1 and the other parameters were defaulted. Then, the ML tree was adjusted for presentation through the interactive tree of life (iTOL, version 6.5.2).

## Supplementary Information

**Additional file 1: Table S1.** The information of α-MF secretion signals using in this study. **Table S2.** Optimized coding sequences of α-mating factor secretion signals. **Table S3.** The information of endogenous signal peptides.

**Additional file 2: Fig. S1.** The construction schematic of expression vectors with different α-MF secretion signal or endogenous signal peptide. The EGFP gene was cloned into pPIC9K between* Sna*B I and* Eco*R I sites. The pPIC9K-EGFP was digested with *Bam*H I and *Sna*B I to remove the α-MF secretion signal of *S. cerevisiae*, and then an α-MF secretion signal from yeast specie or endogenous signal peptide was inserted to replace the α-MF signal leader of *S. cerevisiae*. **Fig. S2.** The structures of pro-peptides of α-MF secretion signals predicted by alphafold2 model.* S.ce: S. cerevisiae*;* W.cl*: *W. ciferrii*; *K.na*: *K. naganishii*; *L.fe*: L. fermentati; *T.bl*: *T. blattae* CBS 6284; *T.ph*: *T. phaffii *CBS 4417; *K.sa*: *K. saulgeensis*; *N.ca*2: *N. castellii *CBS 4309 (alpha 2); *C.al*: *C. albicans *P75063; *S.bo*: *S. boulardii*; *N.ca1*: *N. castellii *CBS 4309 (alpha 1); *L.th: L. thermotolerans* CBS 6340; *N.da1*: *N. dairenensis* CBS 421 (alpha 1); *G.ca*: *G. candidum*; *N.cr*: *N. crass*; *L.me*: *L. meyersii* CBS 8951; *E.cy*: *E. cymbalariae*; *L.da*: *L. dasiensis* CBS 10888; *Y.li*: *Y. lipolytica* CLIB122; *M.fa*: *M. farinosa* CBS 7064; *L.sp*: *Lachancea sp. *CBS 6924; *S.ku*1: *S. kudriavzevii *(alpha 1); *L.qu*: *L. quebecensis*; *C.pa*: *C. parapsilosis*; *A.po*: *A. porosum*; *Z.me*:* Z. mellis*; *L.mi*: *L. mirantina*; *S.pa*: *S. paradoxus*; *L.no: L. nothofagi* CBS 11611; *S.ku2: S. kudriavzevii *(alpha 1); *Z.ro*: *Z. rouxii*; *M.pu*:* M. aff. pulcherrima*; *C.or*:* C. orthopsilosis* Co 90-125; *K.pa*:* K. pastoris*; *N.da*2: *N. dairenensis *CBS 421 (alpha 2); *S.st*: *S. stipitis *CBS 6054; *D.ha: D. hansenii* CBS767; H.op: *H. opuntiae.*

## Data Availability

The data supporting the conclusions of this article are included with the article and its Additional files 1 and 2.
