# Supplementary material for: The α-mating factor secretion signals and endogenous signal peptides for recombinant protein secretion in Komagataella phaffii
Source: Biotechnol Biofuels Bioprod. 2022 Dec 16;15:140. doi: 10.1186/s13068-022-02243-6 (PMC9756452; doi:10.1186/s13068-022-02243-6)
Supplement: Supplementary file 1 — Additional file 1: Table S1. The information of α-MF secretion signals using in this study. Table S2. Optimized coding sequences of α-mating factor secretion signals. Table S3. The information of endogenous signal peptides. [file 13068_2022_2243_MOESM1_ESM.docx]

Table S1. The information of α-MF secretion signals using in this study

| **Species** | **Abbreviation** | **α-factor pre-pro peptides** | **Length** | **Accession number** |
| --- | --- | --- | --- | --- |
| *Apiotrichum porosum* | *A. po* | MKLFAVLVALAASSAVTASPIPNADAAPVAEAQSGWGSSAGGSPSWKR | 48 | RSH86260 |
| *Candida albicans* P75063 | *C. al* | MKFSLTLLTATIATIVAAAPSQYTGQAIDSNQVVEIPESAVEAYFSIDDELTPVFGEIDNKPVILIVNGTILTSGANNEKR | 81 | KGU35989 |
| Candida orthopsilosis Co 90-125 | *C. or* | MKFSITVLTSVAAALVASAPVTPGKIDTPALPIENPLERVVEAFFKGSSIDAEAENKVEDKAVAEAMEDADAKTAAGSQKR | 81 | CCG22545 |
| Candida parapsilosis | *C. pa* | MKFSIAVLTAIAAALVASAPVASKEAEVPALPVDNVLERVVEAFFNGPSIDAEIKDKTAADVKGVVGSQKR | 71 | CCE40685 |
| Debaryomyces hansenii CBS767 | *D. ha* | MKFSIFALTTLVSTISLAAAAPTPDSTETGKYVVSDYLVPDEAINNKVEITDDQQPLVVEESGKKYVLIVNATLAESVISKAGIDIEGLEAAFAKSDDTASVSKR | 105 | CAG89586 |
| Eremothecium cymbalariae | *E. cy* | MKFYNILSVASIASLVFAAPVSVNDAKEIAATFPQEALLGFLDLTDAENIVILSLVDEEKSGIALVNKTIWATARSEQAAGISKR | 85 | ADZ57212 |
| Geotrichum candidum | *G. ca* | MRFSLATVYAFTVIGTVLGVPIASSEPTATTLSTVAAASATFSPGGDSPFTGIKNFPDFASFPPFPPGFDTGLSKR | 76 | CDO51157 |
| *Hanseniaspora opuntiae* | *H. op* | MKFTTAIASVAAAASFATAAAIDQEQLTNGTYIDIPQESILSFLDLTDSPEVSVYPIKEGSKTGLLFVNSTIVDQAYNETTPLTRKR | 87 | OEJ87413 |
| Komagataella pastoris | *K. pa* | MKPLILSAILITLCITSIAVSAPVEGIFADLHDSSHPYITDDVGVRMSAIKEENPDRHFVGDEIPDEAVIQSFKSKRREILFLLDDIGSAVEFIGSNLAQIEANHLSERVQFSWTHIRKNQPFGKR | 126 | ANZ74863 |
| Komagataella phaffii | *K. ph* | MKSLILNIISVTLAITSTAASAPVESIFANQPDSSLTDTNDGVGVGMSTIKEEDFGKHFVENQILDEAVIMSLKLRKGVNLFFLDDIGLATELIGNKIAQIEAIDLSERLAQSWTNIRKNRLFGKR | 126 | AOA62422 |
| Kluyveromyces lactis | *K. la* | MKFSTILAASTALISVVMAAPVSTETDIDDLPISVPEEALIGFIDLTGDEVSLLPVNNGTHTGILFLNTTIAEAAFADKDDLKKR | 85 | XP_454814 |
| *Kazachstania naganishii* | *K. na* | MKFSTILSAAALAASAFAAPIEQPEQDLNSTTIPAEAIISYLDLEGDKDIAVVPFSNATDSGLLFVNTTILAQANKEAGTPLQKR | 85 | BAC00922 |
| Kazachstania saulgeensis | *K.sa* | MKFVNTLSLLATVAVTLAAPVNITETSETSKATIPAEAIIGYLDLEGNKDVALLPFHNETSNGLMFVNTTIINSALAETEDEDLVKR | 87 | SMN18993 |
| Lachancea dasiensis CBS 10888 | *L. da* | MFREFSAYLTLALAMSRAWAAPVNLSSSLSMNEGQSLPAESIVGFLDLTGADDLALFPVSNATNTGVLIVNTTVLDSATESKKRGKR | 87 | SCU84268 |
| Lachancea fermentati | *L. fe* | MRLSSLVTATVFFSTALCLPVRSTNPLPSGTSVPDEAILAFIDLTEDDDVAVVRASNDTNSGLLLVNTTLLAAMEHDHEAQAHKR | 85 | SCW00568 |
| Lachancea meyersii CBS 8951 | *L. me* | MFKTLSIYVALALAAKGVTAAPISTNESSPALPLESISGYLDLTRADDLALLPVSNDTHTGLLVVNTTILAAAMASESTYTKR | 83 | SCU84249 |
| Lachancea mirantina | *L.mi* | MKLTVFWTAFLSFLTTFIVTASAAPVADSANDGSVNIPAESIIGFLDLSDSKDATLVPVSNGTHSGFLFLNSTILSQASTESLKKR | 86 | SCU88438 |
| Lachancea nothofagi CBS 11611 | *L. no* | MYQTINVCLALTLAVTNVCAAPIKFNDSSPALPLESISGYLDLTGAEDLALLPVSNATHTGILVVNTTILASALASESNYNKR | 83 | SCU99144 |
| Lachancea quebecensis | *L. qu* | MLKSVSFFLTLALSSITASAAPINITSSDPAIPSESISGFLDLTDAEDLALLPVSNGTHSGVLIVNTTILAQAFGSDDVLTKR | 83 | CUS23216 |
| Lachancea sp. CBS 6924 | *L. sp* | MFKRFSIYLALALAKLAAAAPITTNDSFPALPQESISGYLDLTGAEDLALLPVSNDTHTGLLVVNTTILASALASENSKQKR | 82 | SCU81073 |
| Lachancea thermotolerans CBS 6340 | *L. th* | MLKSFSLFLAFALTSITVSAAPINITDSDPVIPRESICGFLDLTDAEDLALLPVSNGTHSGILVVNTTILAQAFGSDDILTKR | 83 | CAR30230 |
| Metschnikowia aff. pulcherrima | *M. pu* | MRLLFSFVLATLAVLTVAEAASDLTDLTDNGTLVPLEAVIGGLPIPDDVFPVFNYENNTATVVFLNATILAEAKAHSAENLTKR | 84 | QBM86424 |
| Millerozyma farinosa CBS 7064 | *M. fa* | MKFSAIVISGLLGAGLVKAAPVDSGAKGKYSRTDLIIPDEAIANRYVVGDDEQPVFAEIDNKPVVYIVNTTKAESIVAKSGITLDDLKESYANATKEEEAKNGKR | 105 | CCE80202 |
| Naumovozyma castellii CBS 4309 (alpha 1) | *N. ca* 1 | MKFSSILSTIALFSASTLATDIETEDATMPQVPAEAVIGYLDFDGDNDIAMLPFANATSSGLLFVNTTLVEQANNEEGSISLAKR | 85 | CCC72065 |
| Naumovozyma castellii CBS 4309 (alpha 2) | *N. ca* 2 | MKLSALLSTVALASTSFAAPIDTTASNENLNSTDIPAEAVIGYLDLGSDSDVAMLPFQNSTSNGLLFVNTTIVQQAAQENDDSVGLAKR | 89 | CCC69946 |
| Neurospora crass | *N.cr* | MKFTLPLVIFAAVASATPVAQPNAEAEAQWCRIHGQSCWKVKRVADAFANAIQGMGGLPPRDESGHQPAQVAKRQVDELAGIIALTQEDVNAYYDSLSLQEKFAPSTEEEKKTEKVAKR | 119 | Q01301 |
| *Naumovozyma dairenensis* CBS 421 (alpha 1) | *N. da* 1 | MKFTTILSAITLLSSSAFAEDISTGDAELPQVPAEAVLGYLDFGSDNDIAMLPFANTTSNGLLFVNTTIVEQANEKLENDPSSLTKR | 87 | CCD27058 |
| *Naumovozyma dairenensis* CBS 421 (alpha 2) | *N. da* 2 | MKFSTLLSTAAALASTSLAAPINTTETIDNSSAATSADIPAEAILGYLDLDGDNDIALLPFANATSNGLLFVNTTIVEQATKEQQNGDGSVDLTKR | 96 | CCD26693 |
| Saccharomyces cerevisiae S288C | *S. ce* | MRFPSIFTAVLFAASSALAAPVNTTTEDETAQIPAEAVIGYLDLEGDFDVAVLPFSNSTNNGLLFINTTIASIAAKEEGVSLDKR | 85 | NP_015137 |
| Saccharomyces kudriavzevii (alpha 1) | *S. ku* 1 | MRFSSIITAVFAASSALAAPVNTTSECETVQIPAEAIIGYLDLEGDFDVAVLPFANSTNNGLLFINTTIANLATKEESVPLSKR | 84 | AGW25001 |
| Saccharomyces kudriavzevii (alpha 2) | *S. Ku* 2 | MKVSSMVSMTILAAASVFASSNDDIVQVPAEAVIGYLDLGRDHDIAALPFSNSTTSGLLFVNTTIVNAAEKKRNATLTKR | 80 | AGW25079 |
| Saccharomyces boulardii | *S. bo* | MKFISTFLTFILAAVSVTASSDEDIAQVPAEAIIGYLDFGGDHDIAFLPFSNATASGLLFINTTIAEAAEKEQNTTLAKR | 80 | KQC43953 |
| Saccharomyces paradoxus | *S. pa* | MRFCSVFTAFLFTASSALTAPVNTTTEDETAQIPAEAIIGYLDLEGDFDIAVLPFSNSTNNGLLFINTTIANIAAEEEGVTLNKR | 85 | AGW24990 |
| *Scheffersomyces stipitis* CBS 6054 | *S. st* | MHLRSTAILSAVVFTSVALSAPTSGQNIDIDFPDESIAGAIPLSYDLVPIIGSYQGQNVILIVNSTIAAASEAAASEGKSKR | 82 | ABN67449 |
| Tetrapisispora blattae CBS 6284 | *T. bl* | MKFSTVLSLVSVAASTTLAAPVDTESLYDNSTTVDVPEEAILAFVGFDGTDVALTPFRNETHSGVLFINTTIYESALASEDGTPLVKR | 88 | CCH59351 |
| *Tetrapisispora phaffii* CBS 4417 | *T. ph* | MKLSSVLSTLALTATSAFAAPVSNESVDNASVPAEAIIGYLNFDGANDIALLPFSNSTTSGVMFINTTIAEQAYEEAGVSLSKR | 84 | CCE65043 |
| *Wickerhamomyces ciferrii* | *W. ci* | MQLSLLTSLAIVSTLLGSSFAAPVENINIKDNGNGTSEADVPGTSQGVEFPFAKEAIIEAVSLGNDIAPIVLNDAVYFVNTTTVDKELESKLGKR | 95 | CCH44525 |
| *Yarrowia lipolytica* CLIB122 | *Y. li* | MKFSTIALAAVACLVSAAPAAPVGTGSHGPQSIPEEAIVGGLQGTENEIFVFFNDDESGKQGIAIIDAKKAQEAGFMDPQPDSEVAAGNAKR | 92 | CAG79619 |
| *Zygosaccharomyces mellis* | *Z. me* | MKLSIALGVALGAAAGLTVPVEEVKR | 26 | GCF01605 |
| Zygosaccharomyces rouxii | *Z. ro* | MRLSIALGVTFGAVAGLTAPVEEVKR | 26 | CAR29418 |

The signal peptides (yellow color) predicted by SignalP-5.0 and cleavage site of Kex2 protease (green color) were highlighted in the table.

Table S2. Optimized coding sequences of α-mating factor secretion signals

| **Species** | Sequence |
| --- | --- |
| *A. po* | ATGAAACTTTTTGCTGTTTTGGTGGCATTAGCAGCTTCTTCTGCTGTCACTGCTTCTCCCATACCCAATGCTGATGCTGCTCCTGTTGCTGAAGCTCAAAGTGGCTGGGGCTCTTCTGCTGGTGGTTCTCCTTCATGGAAACGA |
| *C. al* | ATGAAGTTTTCTTTAACTTTACTAACTGCTACCATTGCCACCATTGTTGCTGCTGCTCCTGCTCAATACACTGGCCAAGCCATTGATTCCAATCAAGTGGTGGAAATTCCAGAGTCTGCTGTTGAAGCTTATTTTCCCATTGAAGATGAGCTAACTCCAGTGTTTGGTGAAATTGACAACAAACCAGTCATTCTCATTGTCAATGGCACCACTTTAACCAGTGGTGCCAACAATGAAAAGAGA |
| *C. or* | ATGAAGTTTTCCATCACTGTTCTTACCAGTGTTGCTGCTGCGTTAGTTGCTTCTGCTCCTGTCACTCCTGGCAAGATTGACACTCCTGCTCTTCCCATAGAGAATCCGTTGGAACGAGTGGTGGAAGCGTTTTTCAAAGGCTCTTCCATTGATGCTGAAGCTGAAAACAAAGTTGAAGACAAGGCTGTTGCTGAAGCCATGGAAGATGCTGATGCCAAAACTGCTGCTGGTTCTCAAAAGAGA |
| *C. pa* | ATGAAGTTTTCCATTGCTGTTCTCACAGCAATTGCTGCTGCGTTAGTTGCTTCTGCTCCTGTTGCTTCCAAAGAAGCTGAAGTTCCTGCTCTTCCTGTTGACAATGTTTTGGAACGAGTGGTGGAAGCGTTTTTCAATGGCCCTTCCATTGATGCTGAGATCAAAGACAAGACTGCTGCTGATGTCAAAGGTGTTGTTGGTTCTCAAAAGAGA |
| *D. ha* | ATGAAGTTCAGCATATTTGCTCTTACCACTTTGGTATCCACCATCAGTTTAGCAGCTGCTGCTCCAACTCCTGATTCCACTGAAACTGGAAAGTATGTTGTCAGTGACTATCTGGTTCCTGATGAAGCCATCAACAACAAAGTTGAGATCACTGATGACCAACAACCGTTGGTGGTGGAAGAAAGTGGAAAGAAGTATGTCCTCATTGTCAATGCCACTTTAGCAGAGTCTGTCATCAGCAAAGCTGGTATTGATATTGAAGGGCTGGAAGCTGCGTTTGCCAAAAGTGATGACACTGCTTCTGTTTCCAAACGA |
| *E. cy* | ATGAAGTTTTACAATATTCTTTCTGTTGCTTCCATTGCTTCTTTGGTGTTTGCTGCTCCTGTTTCTGTCAATGATGCCAAAGAAATTGCTGCTACGTTTCCTCAAGAGGCGTTACTAGGATTTTTGGATCTCACAGATGCTGAGAATATTGTCATTCTTTCTTTGGTGGATGAAGAAAAGAGTGGAATAGCGTTAGTCAACAAAACGATATGGGCCACTGCTCGTAGTGAACAGGCTGCTGGTATCAGCAAACGA |
| *G. ca* | ATGCGGTTTTCTTTGGCCACTGTTTATGCTTTCACTGTCATTGGAACTGTTCTTGGTGTTCCAATTGCTTCTTCAGAGCCAACTGCTACCACTCTTTCCACTGTTGCTGCTGCTTCTGCTACGTTTTCTCCTGGTGGTGACTCTCCGTTCACTGGTATCAAGAACTTTCCTGACTTTGCTTCGTTTCCTCCGTTTCCTCCTGGATTTGACACTGGTCTTTCCAAACGA |
| *H. op* | ATGAAGTTTACCACTGCCATTGCTTCTGTTGCTGCTGCTGCTTCGTTTGCCACTGCTGCTGCCATTGACCAAGAGCAACTAACCAATGGAACTTACATTGATATTCCTCAAGAGAGCATTCTTTCATTTTTGGATCTCACAGACTCTCCTGAAGTCAGTGTTTATCCCATCAAAGAAGGCTCCAAAACTGGTCTTTTATTTGTCAACTCCACCATTGTTGACCAAGCGTACAATGAAACCACTCCGTTGACCAGAAAGAGA |
| *K. pa* | ATGAAACCGTTGATTCTTTCTGCCATTCTTATCACTTTATGTATCACTTCCATTGCTGTTTCTGCTCCAGTGGAAGGGATATTTGCTGATCTTCATGACTCTTCTCATCCTTATATCACTGATGATGTTGGTGTTCGTATGAGTGCCATCAAAGAAGAGAATCCTGATCGTCATTTTGTTGGTGATGAAATTCCTGATGAAGCTGTCATTCAATCGTTCAAAAGCAAACGAAGAGAAATTCTTTTCCTTTTAGATGATATTGGCTCTGCTGTTGAGTTCATTGGCTCCAATTTGGCCCAGATTGAAGCCAATCATTTGAGTGAACGAGTTCAGTTCAGTTGGACTCATATCAGAAAGAATCAACCGTTTGGCAAACGA |
| *K. ph* | ATGAAATCTCTGATACTCAATATCATCAGTGTCACTTTGGCCATCACCAGCACAGCAGCTTCTGCTCCAGTGGAAAGCATATTTGCCAATCAACCTGACTCTTCTTTAACTGACACCAATGATGGTGTTGGTGTTGGAATGTCCACCATCAAAGAAGAAGACTTTGGCAAACATTTTGTGGAGAATCAGATTCTTGATGAAGCTGTCATCATGTCTTTGAAACTTCGTAAAGGTGTCAATCTTTTCTTTTTGGATGATATTGGTTTGGCCACTGAGCTGATTGGCAACAAGATTGCTCAAATTGAAGCCATTGATCTCAGTGAAAGGCTGGCTCAATCATGGACCAATATCAGAAAGAATCGTCTTTTTGGCAAACGA |
| *K. la* | ATGAAGTTTTCCACCATTCTTGCAGCTTCCACTGCTCTGATATCAGTTGTCATGGCTGCTCCTGTTTCCACTGAAACTGATATTGATGATCTTCCCATATCAGTTCCAGAGGAAGCGTTGATTGGATTCATTGATCTCACTGGTGATGAAGTTTCTTTACTTCCTGTCAACAATGGAACTCACACTGGTATTCTTTTCCTCAATACCACCATTGCTGAAGCTGCGTTTGCTGACAAAGATGATCTCAAGAAACGA |
| *K. na* | ATGAAGTTTTCCACCATTCTTTCTGCTGCTGCGTTAGCAGCTTCTGCGTTTGCTGCTCCAATTGAACAGCCAGAGCAAGATCTCAACTCCACCACCATTCCAGCAGAGGCCATCATCAGCTATCTTGATTTGGAAGGTGACAAAGATATTGCTGTTGTTCCTTTCAGCAATGCCACTGACTCTGGGCTTTTATTTGTCAATACCACCATTCTTGCTCAAGCCAACAAAGAAGCTGGAACTCCTTTACAGAAACGA |
| *K.sa* | ATGAAGTTTGTCAACACTTTATCGTTACTAGCCACTGTTGCTGTCACTTTAGCAGCTCCTGTCAATATCACTGAAACCAGTGAAACTTCCAAGGCCACCATTCCAGCAGAGGCCATCATTGGCTATCTTGATTTGGAAGGCAACAAAGATGTTGCTCTTCTTCCGTTCCACAATGAAACTTCCAATGGGCTGATGTTTGTCAATACCACCATCATCAACTCTGCTCTTGCTGAAACTGAAGATGAAGATTTGGTGAAACGA |
| *L. da* | ATGTTCAGAGAGTTTTCTGCTTATCTCACTTTAGCGTTGGCCATGTCAAGGGCTTGGGCTGCTCCTGTCAATCTTTCTTCTTCTCTTTCCATGAATGAAGGCCAATCGTTACCAGCAGAGAGCATTGTTGGATTTTTGGATCTCACTGGTGCTGATGATCTTGCTCTTTTCCCTGTTTCCAATGCCACCAACACTGGTGTTCTCATTGTCAATACCACTGTTTTGGACTCTGCTACAGAGAGCAAGAAACGAGGCAAACGA |
| *L. fe* | ATGCGTCTTTCTTCTTTAGTCACTGCTACAGTGTTTTTCAGCACAGCACTCTGTCTTCCTGTTCGTTCCACCAATCCTCTTCCCAGTGGAACTTCAGTTCCTGATGAAGCCATTCTTGCCTTCATTGATCTCACAGAAGATGATGATGTTGCTGTTGTTCGTGCTTCCAATGACACCAACAGTGGGCTTTTACTAGTCAATACCACTTTACTAGCTGCCATGGAACACGATCATGAAGCTCAAGCGCACAAGAGA |
| *L. me* | ATGTTCAAAACTCTTTCCATCTACGTTGCTCTTGCATTAGCTGCCAAAGGTGTCACTGCTGCTCCTATATCCACCAATGAATCGTCTCCTGCTCTTCCGTTGGAAAGCATATCAGGCTATCTTGATCTTACCAGAGCTGATGATCTTGCTCTTCTTCCTGTTTCCAATGACACTCACACAGGGCTTTTAGTTGTCAATACCACCATTCTTGCAGCTGCCATGGCCAGTGAATCAACTTACACCAAACGA |
| *L.mi* | ATGAAACTCACAGTGTTTTGGACTGCGTTTCTTTCATTTTTGACCACTTTCATTGTCACTGCTTCTGCTGCTCCTGTTGCTGACTCTGCCAATGATGGCTCTGTCAATATTCCAGCAGAGTCCATCATTGGATTTTTGGATCTCAGTGATTCCAAAGATGCCACTTTAGTTCCTGTTTCCAATGGAACTCACAGTGGATTTCTTTTCCTCAACTCCACCATTCTTTCTCAAGCTTCCACAGAGTCTTTGAAGAAACGA |
| *L. no* | ATGTATCAGACCATCAATGTTTGCCTGGCGTTAACTTTAGCTGTCACCAATGTTTGTGCTGCTCCAATCAAGTTCAATGACTCTTCTCCTGCTCTTCCGTTGGAAAGCATATCAGGCTATCTTGATCTCACTGGTGCTGAAGATCTTGCTCTTCTTCCTGTTTCCAATGCCACTCACACTGGTATTCTAGTTGTCAATACCACCATTCTTGCTTCTGCTTTGGCCAGTGAAAGCAACTACAACAAACGA |
| *L. qu* | ATGCTCAAATCGGTGTCGTTTTTCCTCACTTTGGCCCTTTCTTCCATCACTGCTTCTGCTGCTCCAATCAATATCACCAGCAGTGATCCTGCCATTCCCAGTGAATCCATCAGTGGATTTTTGGATCTCACAGATGCTGAAGATCTTGCTCTTCTTCCTGTTTCCAATGGAACTCATTCTGGTGTTCTCATTGTCAATACCACCATTCTTGCTCAAGCGTTTGGCTCTGATGATGTTCTTACCAAACGA |
| *L. sp* | ATGTTCAAACGATTCAGCATATATCTTGCCCTGGCGTTGGCCAAACTTGCAGCTGCTGCTCCAATCACCACCAATGATTCGTTTCCTGCTCTTCCTCAAGAGAGCATATCAGGCTATCTTGATCTCACTGGTGCTGAAGATCTTGCTCTTCTTCCTGTTTCCAATGACACTCACACAGGGCTTTTAGTTGTCAATACCACCATTCTTGCTTCTGCTTTGGCCAGTGAAAACTCCAAACAGAAACGA |
| *L. th* | ATGCTCAAATCGTTCAGTCTTTTCCTGGCGTTTGCTTTGACTTCCATCACTGTTTCTGCTGCTCCAATCAATATCACTGACTCTGATCCTGTCATTCCAAGAGAAAGCATATGTGGATTTTTGGATCTCACAGATGCTGAAGATCTTGCTCTTCTTCCTGTTTCCAATGGAACTCACAGTGGAATACTAGTTGTCAATACCACCATTCTTGCTCAAGCGTTTGGCTCTGATGATATTCTTACCAAACGA |
| *M. pu* | ATGCGTCTTCTTTTCAGTTTTGTTCTTGCCACTTTAGCTGTTCTCACAGTGGCAGAGGCTGCCAGTGATCTCACAGATCTCACAGACAATGGAACTTTGGTGCCGTTGGAAGCTGTCATTGGTGGTCTTCCCATACCGGATGATGTTTTCCCAGTGTTCAACTATGAAAACAACACAGCAACAGTGGTGTTTTTGAATGCCACCATTCTTGCAGAGGCCAAAGCTCATTCTGCTGAGAATCTTACCAAACGA |
| *M. fa* | ATGAAGTTTTCTGCCATTGTCATCAGTGGGCTTTTAGGTGCTGGTTTGGTGAAAGCTGCTCCTGTTGACTCTGGTGCCAAAGGCAAATATTCTCGTACTGATCTCATAATACCGGATGAAGCCATTGCCAATCGTTATGTTGTTGGTGATGATGAACAGCCAGTGTTTGCTGAAATTGACAACAAACCAGTGGTGTACATTGTCAATACCACCAAAGCTGAAAGCATTGTTGCCAAATCTGGTATCACTTTAGATGATCTCAAAGAAAGCTATGCCAATGCCACCAAAGAAGAAGAAGCCAAGAATGGCAAACGA |
| *N. ca* 1 | ATGAAGTTTTCTTCCATTCTTTCCACCATTGCTCTTTTCAGTGCTTCCACTTTGGCCACTGATATTGAAACTGAAGATGCTACCATGCCTCAAGTTCCAGCAGAGGCTGTCATTGGCTATTTGGACTTTGATGGTGACAATGATATTGCCATGCTTCCGTTTGCCAATGCCACTTCCAGTGGGCTTTTATTTGTCAATACCACTTTGGTGGAACAGGCCAACAATGAAGAAGGCTCCATTTCTTTGGCCAAACGA |
| *N. ca* 2 | ATGAAACTTTCTGCTCTTCTTTCCACTGTTGCTTTGGCCTCCACTTCATTTGCTGCTCCAATTGACACCACTGCTTCCAATGAGAATCTCAACTCCACTGATATTCCAGCAGAGGCTGTCATTGGCTATCTTGATCTTGGAAGTGACTCTGATGTTGCCATGCTTCCGTTTCAGAATTCCACCAGCAATGGGCTTTTATTTGTCAATACCACCATTGTTCAACAAGCTGCTCAAGAGAATGATGACTCTGTTGGTTTGGCCAAACGA |
| *N.cr* | ATGAAGTTCACTTTACCGTTGGTGATATTTGCTGCTGTTGCTTCTGCCACTCCTGTTGCTCAACCCAATGCTGAAGCTGAAGCTCAATGGTGTCGTATTCATGGCCAATCATGCTGGAAAGTGAAACGAGTTGCTGATGCGTTTGCCAATGCCATTCAAGGAATGGGTGGTCTTCCTCCAAGAGATGAAAGTGGCCACCAGCCTGCTCAAGTTGCCAAACGACAAGTTGATGAGCTTGCTGGTATCATTGCTCTTACCCAAGAAGATGTCAATGCCTACTATGACTCTTTATCGTTACAAGAGAAGTTTGCTCCTTCCACAGAGGAAGAAAAGAAAACAGAGAAAGTTGCCAAACGA |
| *N. da* 1 | ATGAAGTTTACCACCATTCTTTCTGCCATCACTTTACTTTCTTCTTCTGCGTTTGCTGAAGATATCAGCACAGGAGATGCTGAGCTTCCTCAAGTTCCAGCAGAGGCTGTTCTTGGCTATTTGGACTTTGGCTCTGACAATGATATTGCCATGCTTCCGTTTGCCAATACCACCAGCAATGGGCTTTTATTTGTCAATACCACCATTGTGGAACAGGCCAATGAGAAACTAGAGAATGATCCTTCTTCTTTAACCAAACGA |
| *N. da* 2 | ATGAAGTTTTCCACTTTACTTTCCACTGCTGCTGCGTTGGCCTCCACCAGTTTAGCAGCTCCAATCAATACCACTGAAACCATTGACAATTCTTCTGCTGCCACTTCTGCTGATATTCCAGCAGAGGCCATTCTTGGCTATCTTGATCTTGATGGTGACAATGATATTGCTCTTCTTCCGTTTGCCAATGCCACTTCCAATGGGCTTTTATTTGTCAATACCACCATTGTGGAACAGGCCACCAAAGAACAGCAGAATGGTGACGGCTCTGTTGATCTTACCAAACGA |
| *S. ce* | ATGCGGTTTCCTTCCATTTTCACTGCTGTTCTTTTTGCTGCTTCTTCTGCTCTTGCAGCTCCTGTCAATACCACCACTGAAGATGAAACTGCTCAAATTCCAGCAGAGGCTGTCATTGGCTATCTTGATTTGGAAGGTGACTTTGATGTTGCTGTTCTTCCGTTCAGCAACTCCACCAACAATGGGCTTCTTTTCATCAATACCACCATTGCTTCCATTGCTGCCAAAGAAGAAGGTGTTTCTTTGGACAAGAGA |
| *S. ku* 1 | ATGCGTTTCAGCTCCATCATCACTGCTGTGTTTGCTGCTTCTTCTGCTCTTGCAGCTCCTGTCAATACCACCAGTGAATGTGAAACTGTTCAAATTCCAGCAGAGGCCATCATTGGCTATCTTGATTTGGAAGGTGACTTTGATGTTGCTGTTCTTCCGTTTGCCAACTCCACCAACAATGGGCTTCTTTTCATCAATACCACCATTGCCAATTTGGCCACCAAAGAAGAATCGGTTCCTCTTTCCAAACGA |
| *S. Ku* 2 | ATGAAAGTTTCTTCCATGGTTTCCATGACCATTCTTGCAGCTGCTTCTGTGTTTGCTTCTTCCAATGATGATATTGTTCAAGTTCCAGCAGAGGCTGTCATTGGCTATCTTGATCTTGGAAGAGATCATGATATTGCTGCTCTTCCGTTCAGCAACTCCACCACCAGTGGGCTTTTATTTGTCAATACCACCATTGTCAATGCTGCTGAAAAGAAACGAAATGCCACTTTAACCAAACGA |
| *S. bo* | ATGAAGTTTATCAGTACGTTTCTCACATTCATTCTTGCAGCTGTCAGTGTCACTGCTTCCAGTGATGAAGATATTGCTCAAGTTCCAGCAGAGGCCATCATTGGCTATTTGGACTTTGGTGGTGACCATGATATTGCATTTCTTCCGTTCAGCAATGCCACTGCCAGTGGGCTTCTTTTCATCAATACCACCATTGCTGAAGCTGCTGAGAAAGAACAGAATACCACTTTGGCCAAACGA |
| *S. pa* | ATGCGGTTTTGTTCGGTGTTCACTGCGTTTCTTTTCACTGCTTCTTCTGCTTTGACTGCTCCTGTCAATACCACCACTGAAGATGAAACTGCTCAAATTCCAGCAGAGGCCATCATTGGCTATCTTGATTTGGAAGGTGACTTTGATATTGCTGTTCTTCCGTTCAGCAACTCCACCAACAATGGGCTTCTTTTCATCAATACCACCATTGCCAATATTGCTGCTGAAGAAGAAGGTGTCACTTTGAACAAACGA |
| *S. st* | ATGCATCTTCGTTCCACTGCCATTCTTTCTGCTGTTGTTTTCACCAGTGTTGCTCTTTCTGCTCCAACCAGTGGGCAGAATATTGATATTGACTTTCCTGATGAAAGCATTGCTGGTGCCATTCCTCTCAGCTATGACCTGGTTCCAATCATTGGCTCTTATCAAGGCCAGAATGTCATTCTCATTGTCAACTCCACCATTGCTGCTGCCAGTGAAGCTGCTGCCAGTGAAGGCAAAAGCAAACGA |
| *T. bl* | ATGAAGTTTTCCACTGTTCTTTCTTTAGTCAGTGTTGCTGCTTCCACCACTTTAGCAGCTCCTGTTGACACAGAGTCTCTCTATGACAATTCCACCACTGTTGATGTTCCAGAGGAAGCCATTCTTGCATTTGTTGGATTTGATGGAACTGATGTTGCTTTGACTCCGTTTCGCAATGAAACTCATTCTGGTGTTCTTTTCATCAATACCACCATTTATGAGTCTGCTTTGGCCAGTGAAGATGGAACTCCGTTGGTGAAACGA |
| *T. ph* | ATGAAACTTTCTTCTGTTCTTTCCACTTTGGCCCTCACAGCAACTTCAGCGTTTGCTGCTCCTGTTTCCAATGAATCGGTTGACAATGCTTCTGTTCCAGCAGAGGCCATCATTGGCTATCTCAACTTTGATGGTGCCAATGATATTGCTCTTCTTCCGTTCAGCAACTCCACCACCAGTGGTGTCATGTTCATCAATACCACCATTGCTGAACAGGCGTATGAAGAAGCTGGTGTCAGTCTTTCCAAACGA |
| *W. ci* | ATGCAACTTTCTTTACTAACTTCTTTGGCCATTGTTTCCACTTTACTAGGCTCTTCGTTTGCTGCTCCAGTGGAGAATATCAATATCAAAGACAATGGCAATGGCACCAGTGAAGCTGATGTTCCTGGAACTTCTCAAGGTGTTGAGTTTCCGTTTGCCAAAGAAGCCATCATTGAAGCTGTTTCTTTAGGCAATGATATTGCTCCAATTGTTCTCAATGATGCTGTTTATTTTGTCAATACCACCACTGTTGACAAAGAGCTGGAAAGCAAACTTGGCAAACGA |
| *Y. li* | ATGAAGTTTTCCACCATTGCTCTTGCAGCTGTTGCTTGTTTGGTGTCTGCTGCTCCAGCAGCTCCTGTTGGAACTGGCTCTCATGGCCCTCAAAGCATTCCAGAGGAAGCCATTGTTGGTGGTCTTCAAGGAACTGAGAATGAAATATTTGTGTTTTTCAATGATGATGAAAGTGGCAAACAAGGTATTGCCATCATTGATGCCAAGAAAGCTCAAGAGGCTGGTTTCATGGATCCTCAACCTGACTCTGAAGTTGCTGCTGGAAATGCCAAACGA |
| *Z. me* | ATGAAACTTTCCATTGCTCTTGGTGTTGCTCTTGGTGCTGCTGCTGGTCTCACTGTTCCAGTGGAAGAAGTCAAACGA |
| *Z. ro* | ATGCGTCTTTCCATTGCTCTTGGTGTTACGTTTGGTGCTGTTGCTGGTCTCACTGCTCCAGTGGAAGAAGTCAAACGA |

Table S3. The information of endogenous signal peptides

| Protein | Description | Locus tag | Amino acid sequence of signal peptide | D-score | GRAVY Score | Alphatic index |
| --- | --- | --- | --- | --- | --- | --- |
| Protein ROT1 | Required for normal levels of the cell wall 1,6-beta-glucan. Involved in a protein folding machinery chaperoning proteins acting in various physiological processes including cell wall synthesis and lysis of autophagic bodies. | PAS_FragB_0048 | MVLIQNFLPLFAYTLFFNQRAALA | 0.9548 | 1.079 | 126.25 |
| Mucin-like protein | cellulose binding | PAS_FragB_0067 | MLSLTKLFIGIILGAFVAA | 0.988 | 2.084 | 174.74 |
| Thiol oxidase | oxidoreductase activity, acting on a sulfur group of donors, disulfide as acceptorprotein disulfide isomerase activity | PAS_chr1-1_0011 | MRIVRSVAIAIACHCITALA | 0.9885 | 1.620 | 151.50 |
| Sod_Cu domain-containing protein | metal ion binding | PAS_chr1-1_0109 | MKLQLFTLTLSLMASSTLA | 0.9935 | 1.111 | 133.68 |
| Endo-1,3-beta-glucanase | Endohydrolysis of (1->3)- or (1->4)-linkages in beta-D-glucans when the glucose residue whose reducing group is involved in the linkage to be hydrolyzed is itself substituted at C-3 | PAS_chr1-1_0130 | MSFSSNVPQLFLLLVLLTNIVSG | 0.9583 | 1.348 | 156.52 |
| Mannan endo-1,6-alpha-mannosidase | mannan endo-1,6-alpha-mannosidase activity | PAS_chr1-1_0147 | MWILWLLTTVSA | 0.997 | 1.650 | 162.50 |
| Protein disulfide isomerase, | isomerase activity | PAS_chr1-1_0160 | MKILSALLLLFTLAFA | 0.998 | 2.175 | 189.38 |
| Peptidyl-prolyl cis-trans isomerase | PPIases accelerate the folding of proteins. It catalyzes the cis-trans isomerization of proline imidic peptide bonds in oligopeptides | PAS_chr1-1_0267 | MKLLNFLLSFVTLFGLLSGSVFA | 0.9983 | 1.678 | 148.26 |
| Glycosidase | hydrolase activity, hydrolyzing O-glycosyl compounds  transferase activity, transferring glycosyl groups | PAS_chr1-1_0293 | MRPVLSLLLLLASSVLA | 0.9999 | 1.882 | 206.47 |
| GPI-anchored aspartyl protease (Yapsin) | aspartic-type endopeptidase activity | PAS_chr1-1_0379 | MFVIQLAFLCLGVSLTTA | 0.9908 | 1.978 | 151.67 |
| 1,3-beta-glucanosyltransferase | Splits internally a 1,3-beta-glucan molecule and transfers the newly generated reducing end (the donor) to the non-reducing end of another 1,3-beta-glucan molecule (the acceptor) forming a 1,3-beta linkage, resulting in the elongation of 1,3-beta-glucan chains in the cell wall. | PAS_chr1-3_0226 | MFKSLCMLIGSCLLSSVLA | 0.9992 | 1.768 | 143.68 |
| 1,3-beta-glucanosyltransferase | Splits internally a 1,3-beta-glucan molecule and transfers the newly generated reducing end (the donor) to the non-reducing end of another 1,3-beta-glucan molecule (the acceptor) forming a 1,3-beta linkage, resulting in the elongation of 1,3-beta-glucan chains in the cell wall. | PAS_chr1-3_0227 | MLSILSALTLLGLSCA | 0.9922 | 1.988 | 183.12 |
| SCP domain-containing protein | Protein of unknown function, has similarity to Pry1p and Pry3p | PAS_chr1-4_0164 | MRLLHISLLSIISVLTKANAE | 0.9994 | 0.976 | 171.90 |
| Mannan endo-1,6-alpha-mannosidase | Random hydrolysis of (1->6)-alpha-D-mannosidic linkages in unbranched (1->6)-mannans. | PAS_chr1-4_0242 | MWRPLVLVLALLRVLSPAKG | 0.9939 | 1.135 | 170.50 |
| Lectin-like protein with similarity to Flo1p | Protein of unknown function, has similarity to Pry1p and Pry3p | PAS_chr1-4_0584 | MFEKSKFVVSFLLLLQLFCVLGVHG | 0.9954 | 1.392 | 140.00 |
| Peptide hydrolase | Vacuolar aminopeptidase Y, processed to mature form by Prb1p | PAS_chr1-4_0611 | MKYLPLVATLASSALA | 0.9674 | 1.213 | 140.62 |
| Cell wall protein with similarity to glucanases | Glycoside Hydrolase Family 17 | PAS_chr2-1_0052 | MLSTILNIFILLLFIQASLQ | 0.9748 | 1.865 | 200.00 |
| ATPase | ATPase involved in protein import into the ER, also acts as a chaperone to mediate protein folding i | PAS_chr2-1_0140 | MLSLKPSWLTLAALMYAMLLVVVPFAKPVRA | 0.952 | 1.268 | 141.61 |
| exo-1,3-beta-glucanase | Major exo-1,3-beta-glucanase of the cell wall, involved in cell wall beta-glucan assembly | PAS_chr2-1_0454 | MNLYLITLLFASLCSA | 0.9939 | 1.700 | 158.75 |
| Cell wall protein that contains a putative GPI-attachment site | Cell wall protein that contains a putative GPI-attachment site | PAS_chr2-2_0148 | MQFGKVLFAISALAVTALG | 0.9843 | 1.542 | 133.68 |
| SCP domain-containing protein | Protein of unknown function, has similarity to Pry1p and Pry3p and to the plant PR-1 class of pathog | PAS_chr3_0076 | MKLSTNLILAIAAASAVVSA | 0.9931 | 1.550 | 156.50 |
| Integral membrane protein | Integral membrane protein of the endoplasmic reticulum, required for normal content of cell wall bet | PAS_chr3_0120 | MKSSWKIGLFFIAFVVELVSC | 0.9947 | 1.371 | 120.48 |
| Glucosidase II beta subunit | The beta subunit is required for the solubility and stability of the heterodimeric enzyme, and is involved in retaining the enzyme within the endoplasmic reticulum | PAS_chr3_0179 | MLRLLTIGSIAVSLFPASA | 0.9964 | 1.521 | 154.21 |
| Putative GPI-anchored aspartic protease | Aspartic-type endopeptidase activity | PAS_chr3_0394 | MYQALLVLSLICFSSA | 0.9657 | 1.719 | 152.50 |
| Putative GPI-anchored protein | Metallopeptidase activity | PAS_chr3_0517 | MRFINLTITSLLALASRTTA | 0.9927 | 0.870 | 132.00 |
| Carboxypeptidase | Putative serine type carboxypeptidase with a role in phytochelatin synthesis | PAS_chr3_0633 | MKSVIWSLLSLLALSQALT | 0.9954 | 1.305 | 169.47 |
| Aspartic protease | Aspartic protease, attached to the plasma membrane via a glycosylphosphatidylinositol (GPI) anchor | PAS_chr3_0866 | MLVAVALVLLLSTGYA | 0.9745 | 2.231 | 195.00 |
| Glycoprotein | Glycoprotein involved in cell wall beta-glucan assembly | PAS_chr3_0960 | MFWLLVLSLISQALA | 0.997 | 2.000 | 188.67 |
| PA14 domain-containing protein | Cell adhesion | PAS_chr3_1145 | MKFPVPLLFLLQLFFIIATQG | 0.9988 | 1.519 | 148.57 |
| O-glycosylated protein | O-glycosylated protein required for cell wall stability | PAS_chr4_0305 | MKLAALSTIALTILPVALA | 0.9953 | 1.863 | 185.26 |
| Peptidyl-prolyl cis-trans isomerase | Peptidyl-prolyl cis-trans isomerase (cyclophilin) of the endoplasmic reticulum | PAS_chr4_0545 | MNLTLIFTLISLLLGVWS | 0.998 | 1.828 | 189.44 |
| Aspartic protease | Aspartic protease, attached to the plasma membrane via a glycosylphosphatidylinositol (GPI) anchor | PAS_chr4_0584 | MLKDQFLLWVALIASVPVSGVMA | 0.974 | 1.439 | 148.26 |
