# Supplementary material for: The α-mating factor secretion signals and endogenous signal peptides for recombinant protein secretion in Komagataella phaffii
Source: Biotechnol Biofuels Bioprod. 2022 Dec 16;15:140. doi: 10.1186/s13068-022-02243-6 (PMC9756452; doi:10.1186/s13068-022-02243-6)
Supplement: Supplementary file 2 — Additional file 2: Fig. S1. The construction schematic of expression vectors with different α-MF secretion signal or endogenous signal peptide. The EGFP gene was cloned into pPIC9K between SnaB I and EcoR I sites. The pPIC9K-EGFP was digested with BamH I and SnaB I to remove the α-MF secretion signal of S. cerevisiae, and then an α-MF secretion signal from yeast specie or endogenous signal peptide was inserted to replace the α-MF signal leader of S. cerevisiae. Fig. S2. The structures of pro-peptides of α-MF secretion signals predicted by alphafold2 model. S.ce: S. cerevisiae; W.cl: W. ciferrii; K.na: K. naganishii; L.fe: L. fermentati; T.bl: T. blattae CBS 6284; T.ph: T. phaffii CBS 4417; K.sa: K. saulgeensis; N.ca2: N. castellii CBS 4309 (alpha 2); C.al: C. albicans P75063; S.bo: S. boulardii; N.ca1: N. castellii CBS 4309 (alpha 1); L.th: L. thermotolerans CBS 6340; N.da1: N. dairenensis CBS 421 (alpha 1); G.ca: G. candidum; N.cr: N. crass; L.me: L. meyersii CBS 8951; E.cy: E. cymbalariae; L.da: L. dasiensis CBS 10888; Y.li: Y. lipolytica CLIB122; M.fa: M. farinosa CBS 7064; L.sp: Lachancea sp. CBS 6924; S.ku1: S. kudriavzevii (alpha 1); L.qu: L. quebecensis; C.pa: C. parapsilosis; A.po: A. porosum; Z.me: Z. mellis; L.mi: L. mirantina; S.pa: S. paradoxus; L.no: L. nothofagi CBS 11611; S.ku2: S. kudriavzevii (alpha 1); Z.ro: Z. rouxii; M.pu: M. aff. pulcherrima; C.or: C. orthopsilosis Co 90-125; K.pa: K. pastoris; N.da2: N. dairenensis CBS 421 (alpha 2); S.st: S. stipitis CBS 6054; D.ha: D. hansenii CBS767; H.op: H. opuntiae. [file 13068_2022_2243_MOESM2_ESM.docx]

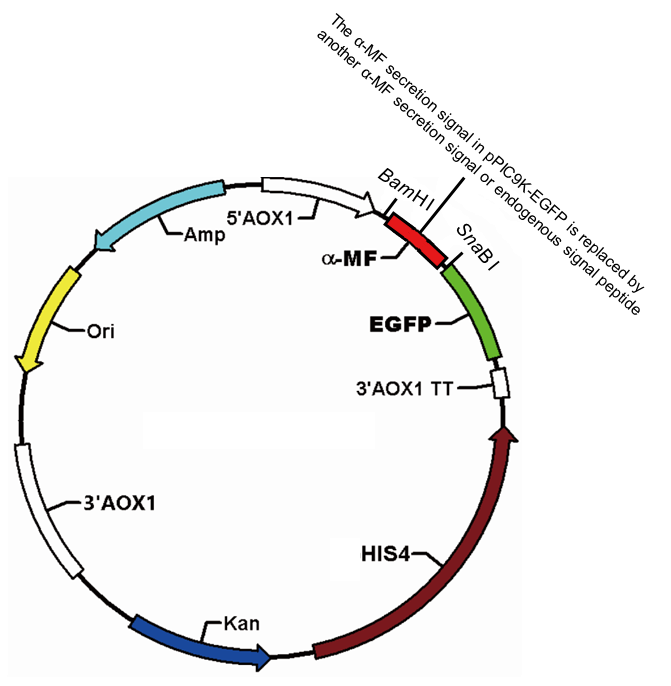


Figure S1. The construction schematic of expression vectors with different α-MF secretion signal or endogenous signal peptide. The EGFP gene was cloned into pPIC9K between *Sna*B I and *Eco*R I sites.The pPIC9K-EGFP was digested with *Bam*H I and *Sna*B I to remove the α-MF secretion signal of *S. cerevisiae*, and then an α-MF secretion signal from yeast specie or endogenous signal peptide was inserted to replace the α-MF signal leader of *S. cerevisiae*.


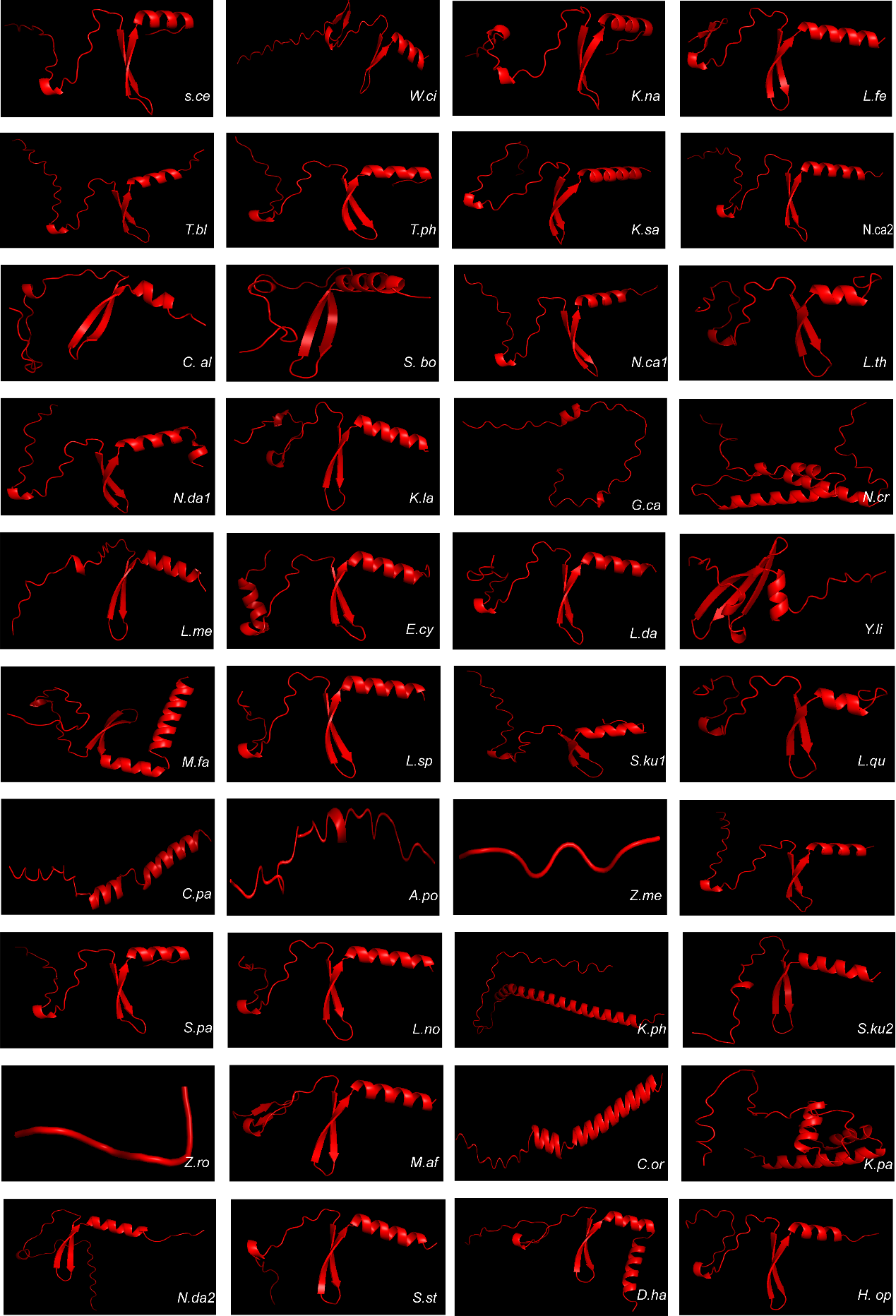


Figure S2. The structures of pro-peptides of α-MF secretion signals predicted by alphafold2 model. *S.ce: S. Cerevisiae*; *W.cl*: *W. ciferrii*; *K.na*: *K. naganishii*; *L.fe*: L. fermentati; *T.bl*: *T. blattae* CBS 6284; *T.ph*: *T. phaffii* CBS 4417; *K.sa*: *K. saulgeensis*; *N.ca*2: *N. castellii* CBS 4309 (alpha 2); *C.al*: *C. albicans* P75063; *S.bo*: *S. boulardii*; *N.ca1*: *N. castellii* CBS 4309 (alpha 1); *L.th: L. thermotolerans* CBS 6340; *N.da1*: *N. dairenensis* CBS 421 (alpha 1); *G.ca*: *G. candidum*; *N.cr*: *N. crass*; *L.me*: *L. meyersii* CBS 8951; *E.cy*: *E. cymbalariae*; *L.da*: *L. dasiensis* CBS 10888; *Y.li*: *Y. lipolytica* CLIB122; *M.fa*: *M. farinosa* CBS 7064; *L.sp*: *Lachancea sp.* CBS 6924; *S.ku*1: *S. kudriavzevii* (alpha 1); *L.qu*: *L. quebecensis*; *C.pa*: *C. parapsilosis*; *A.po*: *A. porosum*; *Z.me*: *Z. mellis*; *L.mi*: *L. mirantina*; *S.pa*: *S. paradoxus*; *L.no: L. nothofagi* CBS 11611; *S.ku2: S. kudriavzevii* (alpha 1); *Z.ro*: Z. rouxii; *M.pu*: *M. aff. pulcherrima*; *C.or*: *C. orthopsilosis* Co 90-125; *K.pa*: *K. pastoris*; *N.da*2: *N. dairenensis* CBS 421 (alpha 2); *S.st*: *S. stipitis* CBS 6054; *D.ha: D. hansenii* CBS767; H.op: *H. opuntiae.*
